# Cell activation-based screening of natively paired human T cell receptor repertoires

**DOI:** 10.1038/s41598-023-31858-4

**Published:** 2023-05-17

**Authors:** Ahmed S. Fahad, Cheng Yu Chung, Sheila N. López Acevedo, Nicoleen Boyle, Bharat Madan, Matías F. Gutiérrez-González, Rodrigo Matus-Nicodemos, Amy D. Laflin, Rukmini R. Ladi, John Zhou, Jacy Wolfe, Sian Llewellyn-Lacey, Richard A. Koup, Daniel C. Douek, Henry H. Balfour, David A. Price, Brandon J. DeKosky

**Affiliations:** 1 Department of Pharmaceutical Chemistry, The University of Kansas, Lawrence, KS 66044 USA; 2 Vaccine Research Center, National Institute of Allergy and Infectious Diseases, National Institutes of Health, Bethesda, MD 20892 USA; 3 Division of Infection and Immunity, Cardiff University School of Medicine, University Hospital of Wales, Cardiff, CF14 4XN UK; 4 Department of Laboratory Medicine and Pathology, University of Minnesota Medical School, Minneapolis, MN 55455 USA; 5 Department of Pediatrics, University of Minnesota Medical School, Minneapolis, MN 55455 USA; 6 Systems Immunity Research Institute, Cardiff University School of Medicine, University Hospital of Wales, Cardiff, CF14 4XN UK; 7 Department of Chemical Engineering, The University of Kansas, Lawrence, KS 66044 USA; 8 Department of Chemical Engineering, Massachusetts Institute of Technology, Cambridge, MA 02142 USA; 9 The Ragon Institute of MGH, MIT, and Harvard, Cambridge, MA 02139 USA

**Keywords:** Biotechnology, Molecular biology

## Abstract

Adoptive immune therapies based on the transfer of antigen-specific T cells have been used successfully to treat various cancers and viral infections, but improved techniques are needed to identify optimally protective human T cell receptors (TCRs). Here we present a high-throughput approach to the identification of natively paired human TCRα and TCRβ (TCRα:β) genes encoding heterodimeric TCRs that recognize specific peptide antigens bound to major histocompatibility complex molecules (pMHCs). We first captured and cloned TCRα:β genes from individual cells, ensuring fidelity using a suppression PCR. We then screened TCRα:β libraries expressed in an immortalized cell line using peptide-pulsed antigen-presenting cells and sequenced activated clones to identify the cognate TCRs. Our results validated an experimental pipeline that allows large-scale repertoire datasets to be annotated with functional specificity information, facilitating the discovery of therapeutically relevant TCRs.

## Introduction

Human T cell receptors (TCRs) mediate cell-based immunity against a variety of cancers and intracellular pathogens via the specific recognition of peptides bound to major histocompatibility complex molecules (pMHCs)^1,2^. Somatic recombination of germline V(D)J gene segments, along with the introduction of junctional variability and subsequent pairing of TCRα and TCRβ chains, yields a highly diverse set of TCRα:β structures^2,3^, which are selected in the thymus on the basis of autologous pMHC reactivity^4^. These processes generate a theoretical diversity of > 10^15^ TCRs and an expressed diversity of ~ 2 × 10^7^ TCRs^5–7^. Moreover, each unique clonotype, defined by the expression of a unique TCR, recognizes a unique array of pMHCs. A detailed analysis of these diverse functional and genetic landscapes is fundamentally important for our molecular understanding of adaptive immunity in health and disease, but it has proven difficult to combine both required sets of information into one experimental pipeline to enable the wholesale discovery of antigen-specific TCRs.

Single-cell analyses are typically required to capture the dual-gene nature of TCRs^7^. A series of techniques have been established to recover natively paired TCRα:β chains, including relative combinatorics-based pairing^7–9^ and single-cell PCR^10–15^, exemplified by the 10x Genomics platform^16^. In addition, several approaches enable the identification and isolation of antigen-specific T cells^7,17^, including the use of recombinant pMHC multimers in conjunction with fluorescence-activated cell sorting (FACS)^18–22^. However, the labile nature of primary T cells precludes a complete interrogation of specificity profiles against multiple pMHCs, and activation-based approaches are further complicated by a lack of effector readouts in the naive pool and functional heterogeneity in the memory pool, including the potential for exhaustion under conditions of persistent antigen stimulation^23^. Robust methods for repertoire-scale functional screening are therefore needed to help understand the connections between antigen specificity and sequence-identified TCRs.

Low-throughput approaches, including single-cell sorting and limiting dilution^24–28^, and even random α:β pairing combined with functional screening of small T cell populations^29^, have been used effectively to discover clinically relevant TCRs. Affinity maturation has also been used to generate therapeutic TCRs, albeit with an attendant risk of serious off-target reactivity against autologous pMHCs^30–32^. Another study reported similar single-cell cloning and coculture techniques to interrogate large-scale libraries comprising natively paired TCRs, but in this case, the engagement of soluble pMHCs was used prior to coculture with donor-matched antigen-presenting cells (APCs)^21^. Due to the important differences between pMHC recognition and T cell activation^33^, as well as the potential difficulties associated with generating recombinant pMHCs for every peptide antigen, a more direct screening method using cell-based coculture could be preferable for mapping functional features of the repertoire of human TCRs. For this purpose, we developed a high-throughput platform incorporating single-cell TCR sequencing and functional screening directly against cell-expressed antigens, using infectious mononucleosis (IM) as a disease model^34–36^. Our workflow comprised native TCR gene cloning into lentiviral display vectors, followed by activation-based screening of the expression libraries in SKW3 cells^37,38^ and the subsequent identification of antigen-specific TCRs via high-throughput sequencing (HTS). The ability to screen thymically selected repertoires comprehensively and repeatedly in this manner should expedite a path to immune discovery and personalized medicine.

## Results

### High-throughput gene capture and screening of native TCRα:β pairs

**Figure 1:**
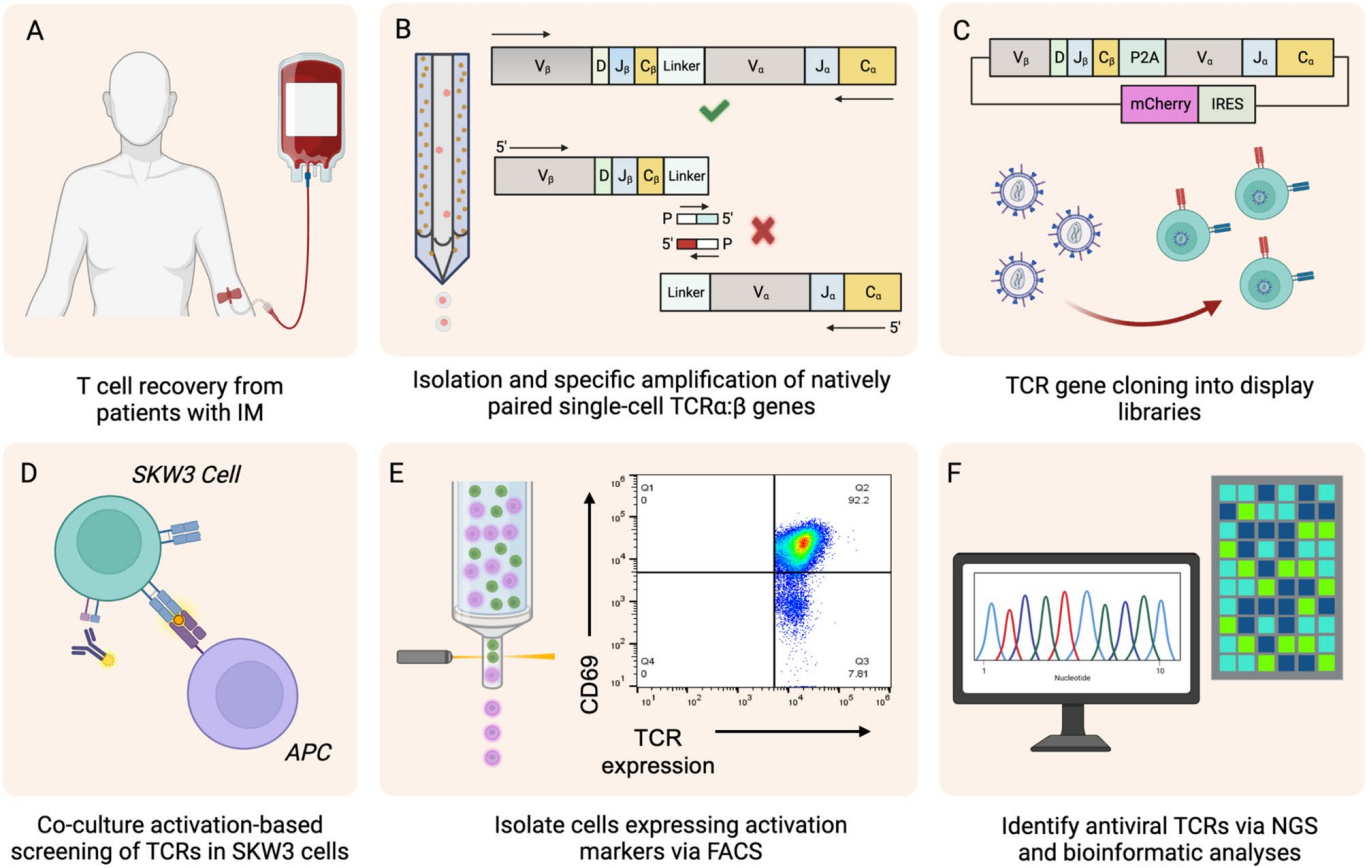
High-throughput TCRα:β gene cloning for cell activation-based screening of human T cell repertoires. (**A**) Peripheral blood mononuclear cells (PBMCs) were recovered from EBV-seropositive donors and expanded briefly in vitro. (**B**) T cells were encapsulated in emulsion droplets with lysis mixture and oligo (dT) beads, and beads with colocalized TCRα and TCRβ mRNAs were subjected to an overlap extension RT-PCR, linking the corresponding variable segments into the same cDNA. A suppression PCR was performed to amplify linked TCRα:β genes in the presence of blocking primers to prevent the association of unpaired TCRα and TCRβ cDNAs. (**C**) Natively paired TCRα:β amplicons were cloned into a lentiviral display construct containing a P2A translation skip motif along with IRES and mCherry elements for mammalian cell expression. TCRα:β plasmids were packaged into lentiviral particles and transduced into SKW3 cells to generate immortalized libraries. (**D**) TCRα:β-SKW3 libraries were cocultured with peptide-pulsed APCs matched to the relevant HLA. (**E**) TCRα:β-SKW3 cells that upregulated CD69 were purified via FACS. (**F**) Purified TCRα:β-SKW3 libraries were characterized via HTS, and computational analysis was used to identify antigen-specific TCRs.

In a previous study, we used an emulsion-based sequencing platform to capture natively paired TCRα:β cDNA amplicons, which were subsequently cloned into expression vectors, displayed in Jurkat cell libraries, and screened using multimeric pMHCs in conjunction with FACS and HTS^34^. This methodology was founded on our established techniques for sequencing, cloning, and screening B cell receptors (BCRs)^39–43^. In the present study, we developed a modified protocol to display natively paired TCRα:β libraries in SKW3 cells, enabling activation-based screening and the characterization of functionally optimal TCRs. The natively paired TCRα:β amplicons interrogated here were obtained previously from two individuals with IM (Fig. 1A)^34^. Briefly, we used a flow-focusing device in the earlier study to encapsulate single T cells inside emulsion droplets containing poly-dT-coated magnetic beads to capture polyadenylated mRNAs (Fig. 1B)^34,40,41,43–45^. Magnetic beads with colocalized TCRα and TCRβ mRNAs were then recovered and used as templates in an overlap extension RT-PCR that physically linked single-cell-derived TCRα and TCRβ genes (Fig. 1B)^34,46^. The resulting TCRα:β libraries were PCR-amplified in the current study to add restriction enzyme sites for cloning into a lentiviral expression vector and subsequently transduced into SKW3 cells (Fig. 1C,D) for activation-based screening via FACS and HTS to link functionality with the gene sequences of individual TCRs (Fig. 1E,F).

### Evaluation of restriction enzyme cloning sites for TCRα:β genes

**Figure 2:**
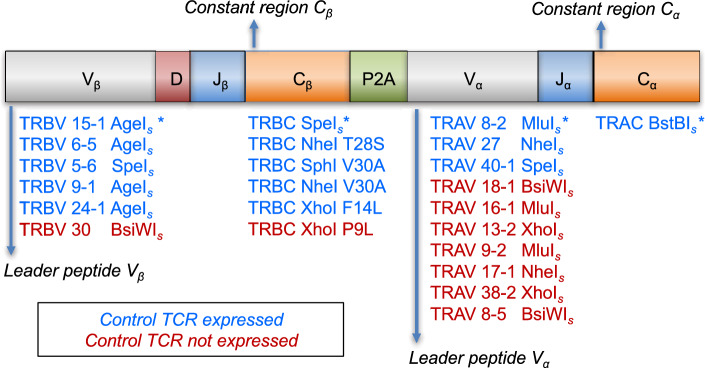
Validation of restriction enzyme site mutations in the TCRα and TCRβ genes. Colors indicate functional mutations (blue) and non-functional mutations (red). *, restriction enzyme site incorporated in the final primer design as reported previously^34^; *s*, silent mutation. Details of each sequence modification are provided in Supplementary Tables 1 and 2.

**Figure 3:**
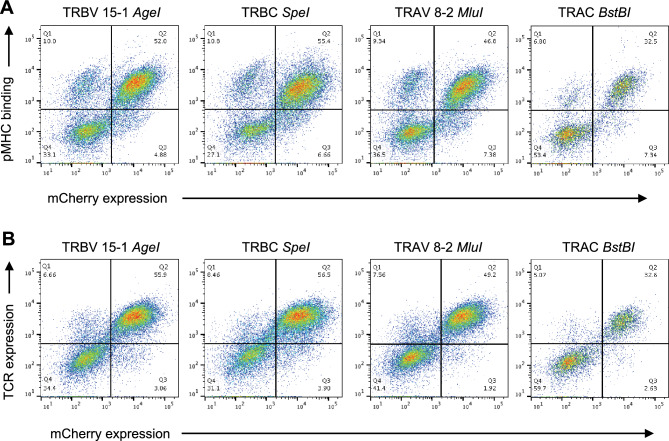
Functionality of restriction enzyme site mutations in the TCRα and TCRβ genes. (**A**) Functional expression of the TM9 TCR cloned via the indicated restriction enzyme sites was tested via flow cytometry using tetrameric complexes of *RM9*/HLA-B*07:02. Internal mCherry expression is shown on the x-axis, and pMHC binding is shown on the y-axis. (**B**) Functional expression of the TM9 TCR cloned via the same restriction enzyme sites was further tested via flow cytometry using anti-human TCRα/β. Internal mCherry expression is shown on the x-axis, and TCR expression is shown on the y-axis. Comparative data from less successful restriction enzyme site mutations are summarized in Supplementary Table 3.

We tested and validated a set of silent and non-silent mutations that introduced restriction enzyme sites into the TCRα and TCRβ variable leader and constant regions (Fig. 2, Supplementary Tables 1 and 2). The effects of these mutations were evaluated in a mammalian lentiviral display system (Fig. 3A,B). Expression constructs encoding a single TCR (TM9) specific for the human leukocyte antigen (HLA)-B*07:02-restricted HIV-1 Nef epitope RPQVPLRPM (*RM9*) were packaged into lentiviral particles and transduced into J.RT3-T3-5/CD8^+^ (J.RT3/CD8) cells, which were subsequently purified on the basis of mCherry expression via FACS. A monoclonal anti-human TCR antibody and fluorescently labeled tetrameric complexes of *RM9*/HLA-B*07:02 were used to quantify expression of the TM9 TCR. Four optimal mutations were selected for inclusion in the final expression construct to enable direct and efficient library-scale cloning of HTS-captured TCRs (Fig. 3 and Supplementary Fig. 1).

### Generation of TCRα:β libraries expressed in SKW3 cells

**Figure 4:**
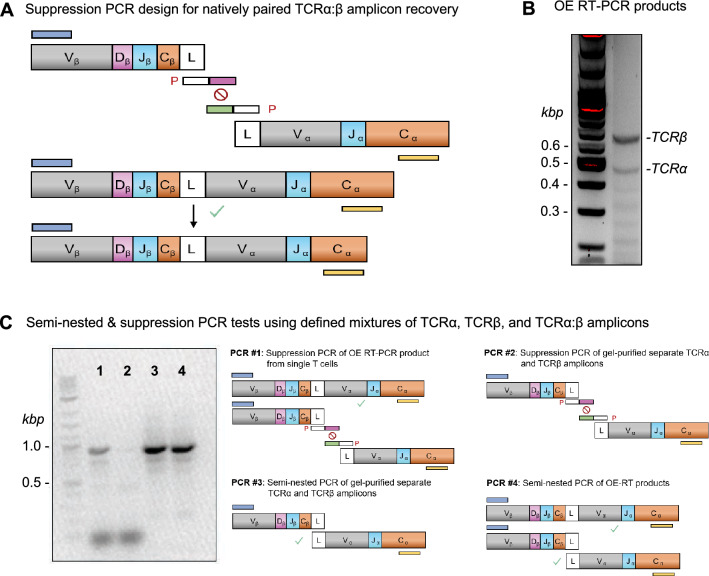
Elimination of non-native TCRα:β pairs via suppression PCR. (**A**) The suppression PCR strategy used to amplify natively paired TCRα:β cDNAs. (**B**) Agarose gel electrophoresis image showing amplification of unpaired TCRα and TCRβ cDNAs via overlap extension (OE) RT-PCR. Left, size ladder; right, experimental lane. (**C**) Agarose gel electrophoresis image comparing products from the first semi-nested PCR with products from various control PCRs. Original gels are presented in Supplementary Fig. 3.

TCRα:β amplicons incorporating the optimal mutations were cloned into the lentiviral pLVX-EF1α-IRES-mCherry vector as reported previously^34^. Briefly, we performed an overlap extension RT-PCR incorporating multiplex primers to amplify and fuse single-cell-derived TCRα and TCRβ chains via a linker sequence^34,46^. We also used a suppression PCR to prevent the random association of unfused TCRα and TCRβ amplicons during the bulk semi-nested PCR (Fig. 4A)^10^.

As expected, agarose gel electrophoresis revealed dominant unlinked TCRα and TCRβ chain bands after the overlap extension RT-PCR (Fig. 4B), which required specific amplification of the subdominant linked TCRα:β amplicons in the mixture. In contrast to our previous fully nested PCR strategies to amplify linked heavy and light chains from expressed BCRs^34,39,41–44^, our TCR cloning strategy was based on unidirectional expression, introducing the potential for unlinked TCRα and TCRβ chains to associate randomly during the bulk semi-nested PCR. To mitigate this issue, we tested a suppression PCR^10^. This approach uses blocking oligonucleotides encoding nonsense sequences at the 5’ ends to prevent amplified single TCRα and TCRβ cDNAs from associating via overlap extension by eliminating sequence homology without affecting natively paired TCRα:β amplicons previously linked in the single-bead emulsion (Fig. 4A–C).

We validated this strategy using a series of control PCRs. Similar amounts of linked TCRα:β material were detected using either unlinked TCRα and TCRβ amplicons (Fig. 4C, *PCR #3*) or fully linked TCRα:β genes (Fig. 4C, *PCR #4*) as templates in standard semi-nested PCRs. In the presence of blocking primers, however, the overlap extension RT-PCR product was amplified successfully (Fig. 4C, *PCR #1*), whereas unfused amplicons no longer yielded a linked TCRα:β product (Fig. 4C, *PCR #2*). We also compared the prevalence of EBV-specific TCRs from our two donors after cloning the corresponding HTS-based libraries into J.RT3/CD8 cells using either the standard semi-nested PCR or the suppression PCR^34^. In line with our expectations based on the elimination of non-natively paired TCRs, the frequencies of J.RT3/CD8 cells that bound the cognate pMHC tetramer were an order of magnitude higher after cloning with the suppression PCR (Supplementary Fig. 2).

### Activation-based screening of TCRα:β expression libraries

TCRα:β amplicon libraries were cloned into the lentiviral vector expression system with all the required elements for full-length TCRα:β expression on the cell surface^34^, including an internal ribosomal entry site (IRES) and an mCherry marker to detect successful transduction via FACS. After cloning the amplified TCRα:β genes into the lentiviral vector backbone using AgeI and BstBI, the linker region was swapped using MluI and SpeI to incorporate a linear DNA construct containing the remaining portions of the TCRβ constant region, a ribosomal skip teschovirus-derived sequence (P2A), and a modified TRAV8-2 leader sequence incorporating an MluI site (Fig. 2, Supplementary Fig. 4) for TCRα expression^34,47^. We generated at least 10^6^ transformants in each cloning step to maintain library diversity (Supplementary Figs. 5 & 6). The full-length TCRα:β expression plasmids were then packaged into lentiviral particles and transduced into SKW3 cells for functional evaluation, illustrated here with reference to Donor 1.

**Figure 5:**
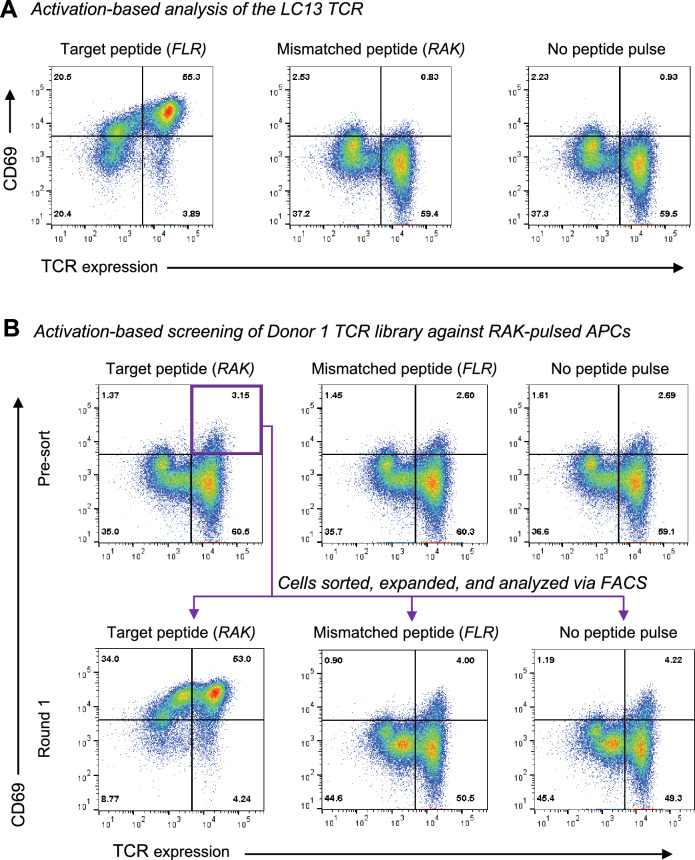
Cell activation-based screening of antigen-specific TCRs. (**A**) Activation of SKW3 cells expressing the LC13 TCR was measured after coculture with *FLR*-pulsed APCs (left), *RAK*-pulsed APCs (center), or unpulsed APCs (right). (**B**) TCRα:β-SKW3 cells from the Donor 1 library were enriched for CD69 expression via FACS after coculture with *RAK*-pulsed APCs (left). Peptide specificity was confirmed using *FLR*-pulsed APCs (center) and unpulsed APCs (right). TCR expression is shown on the x-axis, and CD69 expression is shown on the y-axis.

TCRα:β-SKW3 libraries expressing mCherry were expanded after purification via FACS. In contrast to primary T cells, these immortalized libraries can be screened over multiple rounds of panning against APCs. T2 cells transduced to express HLA-B*08:01 (T2-B8) were pulsed with the EBV BZLF1 peptide RAKFKQLL (*RAK*) at a concentration of 1 mM and cocultured with mCherry^+^ TCRα:β-SKW3 cells for 24 h. Antigen-specific TCRα:β-SKW3 cells were detected via upregulation of the activation marker CD69 (Fig. 5). Coculture parameters were optimized using the EBV EBNA3A-specific LC13 TCR, which recognizes the FLRGRAYGL (*FLR*) epitope restricted by HLA-B*08:01 (Fig. 5A)^48^. *RAK*-specific TCRα:β-SKW3 cells were enriched > 10-fold compared with the presort libraries after a single round of purification via FACS (Fig. 5B). In addition, there was minimal background activation and minimal reactivity against *FLR*-pulsed T2-B8 cells, despite initial coculture in the presence of donor-mismatched MHCs (Fig. 5B).

### Rapid bioinformatic detection of antigen-specific TCRs

HTS-based library analyses were used to identify *RAK*-specific TCRs. Data were processed as reported previously^34^. Briefly, raw FASTQ files were quality-filtered and annotated using MIXCR^49^. Out-of-frame V(D)J reads were excluded from the dataset, and productive in-frame reads were paired by Illumina ID. Reads were compiled using CDR3 and VJ gene identity. CDR3β sequences were clustered to 96% nucleotide identity after excluding singleton reads to minimize errors introduced via HTS and/or PCR. CDR3β amino acid sequences were used to track TCR clones^42^. The frequency of each individual clone in each sorted sample was calculated to evaluate the functional performance of each library. We also calculated the enrichment ratio for each CDR3β. Two known antigen-specific TCR clones identified by sequence analysis were enriched > 10-fold compared with the parental mCherry^+^ TCRα:β-SKW3 library and comprised the bulk of the response against cell-displayed *RAK*/HLA-B*08:01 (Supplementary Table 4).

## Discussion

T cell immunotherapy holds great promise for the treatment of various cancers and infectious diseases, but comprehensive molecular platforms are required to characterize the antigen specificity, functionality, and translational potential of individual TCRs. In this study, we developed and validated a high-fidelity cloning strategy to enable activation-based screening of natively paired TCRα:β gene libraries against peptide-pulsed APCs. These efforts allowed us to link the efficacy of signal transduction in response to cognate antigen encounter with sequence information across the somatically rearranged genome via an integrated experimental and bioinformatics pipeline, facilitating the discovery of naturally selected and optimally potent TCRs.

Our screening technology was adapted from previous work that enabled the physical identification of antigen-specific TCRα:β clones based on the engagement of multimeric pMHCs^34^. We report here the design and validation of mutations enabling the introduction of restriction enzyme sites for high-throughput cloning of TCRα:β genes into mammalian display vectors and the elimination of randomly unassociated TCRα and TCRβ genes via a suppression PCR. The latter greatly enriched our libraries for natively paired TCRs^10^. Although other methods can be used to enhance the fraction of TCRα:β genes^39–43^, the incorporation of a suppression PCR in our workflow proved to be compatible with the use of standard in-line cassettes for gene expression, harmonizing with established methods for single-cell isolation^15,29,50–53^ and microfluidic encapsulation^16,54^. Moreover, our libraries were genetically diverse, with each containing an average 17,241 unique clones after stringent quality filtering and clustering of homologous TCRs.

Importantly, our approach enabled the facile cloning of physically linked TCRα:β genes into various display systems, including Jurkat cells, which can be screened using multimeric pMHCs^34^, and SKW3 cells, which can be screened using the activation-based method reported here. Such immortalized/renewable TCRα:β library screening techniques will be essential for the analysis of peptide specificity, MHC restriction, and the biophysical properties of the corresponding TCRs, which ultimately govern T cell behavior in vivo. However, we found it difficult to barcode cells for the inclusion of singleton TCRα:β genes, which compromised our ability to calculate the efficiency of recovery via HTS. We are currently planning to resolve these issues by incorporating single-cell barcoding techniques and to extend the scope of our work by recovering information on gene transcription and protein expression in association with individual TCRs.

Importantly, our SKW3 cell expression system allowed us to screen TCRα:β gene libraries functionally, measuring responsiveness via the upregulation of CD69. No such activation was observed using Jurkat cell lines in our earlier study^34^. This advance is critically important for immune discovery, because the ability to deliver an activation signal is not equivalent across all antigen-specific TCRs^33^. It can also be difficult in some cases to produce large quantities of soluble pMHCs^21,29,55^. A key feature of our approach was the standardized assessment of functionality using an immortalized cell line. Library screening on this basis can eliminate potential bias arising from the heterogeneity of primary T cells, although it should be noted that other effector readouts may also afford high levels of sensitivity^56,57^. In addition, our method could be adapted for use with cancerous or infected cells rather than peptide-pulsed APCs. Accordingly, it should be feasible to screen for reactivity against target cells presenting biologically relevant densities of disease-associated pMHCs, thereby enhancing the discovery of protective and translationally efficacious TCRs.

There were some limitations to our approach. In particular, the gene capture and sequencing process revealed that repertoire diversity was reduced to around tens of thousands of TCRα:β clonal clusters after excluding singletons in each library, which are more error-prone than TCRα:β clones observed more than once). The degree of loss from the native repertoire was difficult to quantify, because our native immune libraries contained an undetermined number of TCRα:β clones, and our conservative bioinformatic filtering excluded singletons that could represent *bona fide* TCR clones. The natural occurrence of restriction enzyme sites used in the cloning process also likely resulted in the destruction of a small fraction of TCRs (estimated based on prevalence at < 1%). It is further notable that we transduced our TCRα:β libraries into SKW3 cells using viral particles at a very low exposure frequency, estimated in the region of 1–5%. This approach was designed to ensure that each cell integrated only one TCR. Our future efforts will focus on the use of CRISPR-targeted TCRα:β engineering to improve efficacy by integrating TCR genes at defined sites, which was recently demonstrated to be highly effective in similar activation-based screening assays for the analysis of libraries displaying synthetically generated TCRs^58^.

In summary, we have developed a high-throughput platform for the identification of functionally responsive antigen-specific TCRs. Our workflow builds substantially on previous reports that linked somatically rearranged gene sequences with antigen specificity via library screening against soluble pMHCs^34^. In particular, we anticipate that molecular-scale functional screening will accelerate bench-to-bedside immune discovery, facilitating the clinical delivery of personalized therapies for various diseases via the rapid isolation of naturally selected and highly potent antigen-specific TCRs.

## Methods

### Introduction of restriction enzyme cloning sites

A monoclonal TCR (TM9) specific for the HLA-B*07:02-restricted HIV-1 Nef epitope *RM9*^59^ was expressed in the lentiviral vector pLVX-EF1α-IRES-mCherry (Takara Bio, Mountain View, CA) to evaluate the functional performance of restriction enzyme cloning site mutations (Supplementary Tables 1 and 2). Leader sequences were modified for TRAV and TRBV. Restriction enzyme sites were introduced as detailed in Supplementary Tables 1 and 2. Expression of the TM9 TCR was quantified via flow cytometry using anti-human TCRα/β–Alexa Fluor 488 (clone IP26; BioLegend, San Diego, CA) and fluorescently labeled tetrameric complexes of *RM9*/HLA-B*07:02.

### Human samples and cell culture

Donor 1 presented with high fever, fatigue, body aches, and headache, with a maximum illness severity of 3, as described previously^34,35^. Donor 2 presented with fever, tender cervical lymph nodes, sore throat, and fatigue. These donors were enrolled in a prospective study of primary EBV infection at the University of Minnesota (IRB 0608M90593)^35^. Venous blood samples were processed via density gradient centrifugation over ACCUSPIN System-Histopaque-1077 (Sigma-Aldrich, St. Louis, MO) to collect PBMCs, which were subsequently cryopreserved at 1 × 10^7^ cells/mL in heat-inactivated fetal bovine serum (Thermo Fisher Scientific, Waltham, MA) containing 10% dimethyl sulfoxide (Sigma-Aldrich, St. Louis, MO). PBMCs were thawed and density-adjusted to 0.5 × 10^6^ cells/mL in complete CTS OpTmizer T Cell Expansion SFM (Thermo Fisher Scientific, Waltham, MA) supplemented with 5% CTS Immune Cell SR (Thermo Fisher Scientific, Waltham, MA), 200 IU/mL IL-2 (National Cancer Institute Preclinical Biologics Repository, Frederick, MD), and 25 μL/mL ImmunoCult Human CD3/CD28 T Cell Activator (STEMCELL Technologies, Cambridge, MA). Cells were expanded in RPMI 1640 medium (Thermo Fisher Scientific, Waltham, MA) containing 10% heat-inactivated fetal bovine serum (Thermo Fisher Scientific, Waltham, MA), 200 IU/mL IL-2 (National Cancer Institute Preclinical Biologics Repository, Frederick, MD), and 25 μL/mL ImmunoCult Human CD3/CD28 T Cell Activator (STEMCELL Technologies, Cambridge, MA) for 7–10 days and then subjected to single-cell emulsification and overlap extension RT-PCR.

### Generation of natively paired TCRα:β expression libraries

TCRα:β cDNA libraries generated in a previous study^34^ were amplified and modified to incorporate restriction enzyme sites using a two-step, semi-nested PCR. An initial semi-nested suppression PCR incorporating blocking oligonucleotides complementary to the 3’ ends of the unfused TCRα and TCRβ products was performed using a HotStart GoTaq Polymerase System (Promega, Madison, WI). A second semi-nested PCR was then performed using a KAPA HiFi HotStart PCR Kit (Roche, Basel, Switzerland). PCR products were recovered using agarose gel electrophoresis and purified using a 1.5% SYBR Safe Agarose Gel (Thermo Fisher Scientific, Waltham, MA).

PCR products were cloned into a modified version of the commercially available pLVX-EF1α-IRES-mCherry Vector (Takara Bio, Mountain View, CA). TCR amplicons and the expression vector were digested with BstBI and AgeI, and the digestion products were gel-purified and ligated using T4 DNA Ligase (New England Biolabs, Ipswich, MA). Ligation products were purified using a DNA Clean & Concentrator Kit (Zymo Research, Irvine, CA) and transformed via electroporation into competent MegaX DH10B T1 Electrocomp Cells (Thermo Fisher Scientific, Waltham, MA). Plasmids were purified using a ZymoPURE II Plasmid Maxiprep Kit (Zymo Research, Irvine, CA). The rest of the expression cassette was then introduced as an insert between the variable regions of the TCRβ and TCRα genes using SpeI and Mlul (New England Biolabs, Ipswich, MA). The insert contained the remaining portion of the TCRβ constant region, a P2A translation skip motif, and a modified version of the TCRα leader peptide sequence containing an MluI site (Fig. 2) to enable full expression of the corresponding heterodimeric TCRs^34^.

### Lentiviral transduction of SKW3 cells

Lentiviruses were packaged using an adherent 293FT cell line (Thermo Fisher Scientific, Waltham, MA). Cells were grown to a confluency of 70–90% in T75 culture flasks (Thermo Fisher Scientific, Waltham, MA) and transfected using Lipofectamine 3000 Transfection Reagent (Thermo Fisher Scientific, Waltham, MA). A master mix was prepared for each library by incubating 8 μg of PSPAX (Addgene, Watertown, MA), 2 μg of PMD2G (Addgene, Watertown, MA), 40 μL of P3000 Reagent (Thermo Fisher Scientific, Waltham, MA), and 12 μg of the pLVX-EF1α-IRES-mCherry plasmid (Takara Bio, Mountain View, CA) for 5 min in 1 mL of Opti-MEM Reduced Serum Medium per T75 flask (Thermo Fisher Scientific, Waltham, MA). Lipofectamine 3000 Transfection Reagent was diluted 1:25 in 1 mL of Opti-MEM Reduced Serum Medium (Thermo Fisher Scientific, Waltham, MA). The diluted transfection reagent was then added to the master mix and incubated for 15–20 min at room temperature. The resultant mixture was added dropwise to the flask containing 293FT cells and incubated for 3 days at 37 °C. Supernatants containing lentivirus were centrifuged at 800 × *g* for 10 min and added at a 3:1 ratio to Lenti-X Concentrator (Takara Bio, Mountain View, CA) in polypropylene centrifuge tubes (50 mL; Thermo Fisher Scientific, Waltham, MA). Lentivirus-concentrator mixtures were incubated overnight at 4 °C and then centrifuged at 1,500 × *g* for 45 min at 4 °C. The pellets were resuspended in 1 mL of RPMI 1640 medium and stored in two aliquots at − 80 °C. For each library transduction, 3 × 10^6^ unmodified SKW3 cells (DSMZ, Braunschweig, Germany) were seeded in 3 mL of RPMI 1640 medium in a single well of a 6-well tissue culture plate (Thermo Fisher Scientific, Waltham, MA), and 400 μL of rapidly thawed lentivirus stock was added in the presence of polybrene at a final concentration of 4 μg/mL (Sigma-Aldrich, St. Louis, MO). Cells were incubated overnight at 37 °C with 5% CO_2_. After transduction, cells were centrifuged at 500 × *g* for 5 min, resuspended in 10 mL of prewarmed RPMI 1640 medium, transferred to T25 culture flasks, and incubated for 3 days at 37 °C with 5% CO_2_. Cells were then washed twice with PBS and sorted for internal mCherry expression via FACS.

### Functional screening assays in SKW3 cells

TCRα:β-SKW3 libraries were cocultured with peptide-pulsed APCs matched to the relevant HLA. Purified mCherry^+^ TCRα:β-SKW3 cells were expanded in vitro, resuspended in fresh RPMI 1640 medium, and seeded at 1 × 10^6^ cells/well/mL in 6-well tissue culture plates (Thermo Fisher Scientific, Waltham, MA). APCs were washed twice with PBS, resuspended at 1 × 10^6^ cells per condition in 100 µL of FACS buffer (PBS containing 0.05% BSA and 2 mM EDTA), and pulsed with the relevant peptides for 4 h at 37 °C. T2-B8 cells (a kind gift from Scott Burrows, QIMR Berghofer) were used to present epitopes restricted by HLA-B*08:01. APCs were then incubated with individual TCRα:β-SKW3 libraries overnight at 37 °C with 5% CO_2_. TCRα:β-SKW3 cells were recovered after stimulation and stained with anti-CD69–FITC (clone FN50; BioLegend, San Diego, CA).

### PCR-based recovery of TCRβ genes

CD69^+^ TCRα:β-SKW3 cells were sorted via FACS. For molecular analysis of each library, mRNA was extracted from 2 × 10^6^ purified TCRα:β-SKW3 cells using a Direct-zol RNA Kit (Zymo Research, Irvine, CA). TCRβ VDJ regions were amplified using a set of primers targeting the modified TRBV15-1 leader region and TRBC. RT-PCR was performed using SuperScript III Reverse Transcriptase and Platinum Taq (Thermo Fisher Scientific, Waltham, MA). A second primer-extension PCR contained adaptors to add a unique molecular identifier to each sample. cDNA amplicons were run on 1.5% agarose gels, and bands at ~ 450 bp were purified using a Gel DNA Recovery and DNA Clean & Concentrator Kit (Zymo Research, Irvine, CA). Libraries were sequenced using a 2 × 300 MiSeq System (Illumina, San Diego, CA).

### Bioinformatic analysis

Our bioinformatics pipeline was designed to ensure high-quality data by reducing sequence errors introduced via HTS and/or PCR^34,39–44^. First, raw sequences were quality-filtered to retain only those with a Phred Quality Score of 20 (i.e., 99% base call accuracy) in at least 50% of the reads using Fastxtoolkit/0.0.14 (http://hannonlab.cshl.edu/fastx_toolkit/). Next, V, D, and J gene annotations were performed using MiXCR/v2.1.12^49^. Out-of-frame V(D)J combinations were excluded from the dataset, and productive in-frame junction sequences were paired by Illumina read ID. Reads were then compiled based on CDR3 nucleotide exact-match sequences and V(D)J gene identity. CDR3β sequences were clustered to 96% nucleotide identity (ignoring terminal gaps) using USEARCH/v5.2.32^60^, and only clusters with ≥ 2 TCRα:β nucleotide reads were included in the final dataset. Last, full-length TCRα:β sequences were recreated by stitching together the CDR3α or CDR3β sequences with the respective TRAV or TRBV genes, which had been mapped using the International ImMunoGeneTics (IMGT) Information System database (library imgt.202141-1)^61^.

### Ethics statement

Human subjects research was approved by the University of Minnesota Institutional Review Board. Informed consent was obtained from all donors with permission to use samples for research investigations according to the principles of the Declaration of Helsinki.

## Supplementary Information

Supplementary Information.

## Data Availability

Raw TCRα:β MiSeq data have been deposited in the NCBI Sequence Read Archive (SRA) under accession number PRJNA827461. Bioinformatics scripts are accessible via GitHub (https://github.com/dekoskylab/T-cell-screening).
