# Supplementary material for: Cell activation-based screening of natively paired human T cell receptor repertoires
Source: Sci Rep. 2023 May 17;13:8011. doi: 10.1038/s41598-023-31858-4 (PMC10192375; doi:10.1038/s41598-023-31858-4)
Supplement: Supplementary file 1 — Supplementary Information. [file 41598_2023_31858_MOESM1_ESM.docx]

**SUPPLEMENTARY FIGURES**


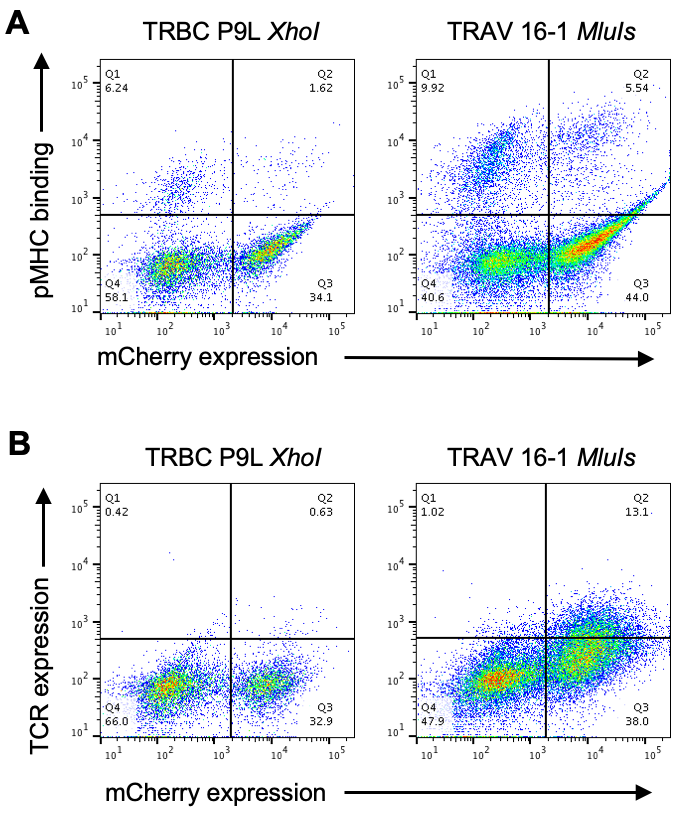


**Supplementary Figure 1. Examples of non-functional cloning site mutations.** (**A**) Functional expression of the TM9 TCR cloned via the indicated restriction enzyme sites was tested via flow cytometry using tetrameric complexes of *RM9*/HLA-B*07:02. Internal mCherry expression is shown on the x-axis, and pMHC binding is shown on the y-axis. (**B**) Functional expression of the TM9 TCR cloned via the same restriction enzyme sites was further tested via flow cytometry using anti-human TCRα/β. Internal mCherry expression is shown on the x-axis, and TCR expression is shown on the y-axis. *s*, silent mutation.


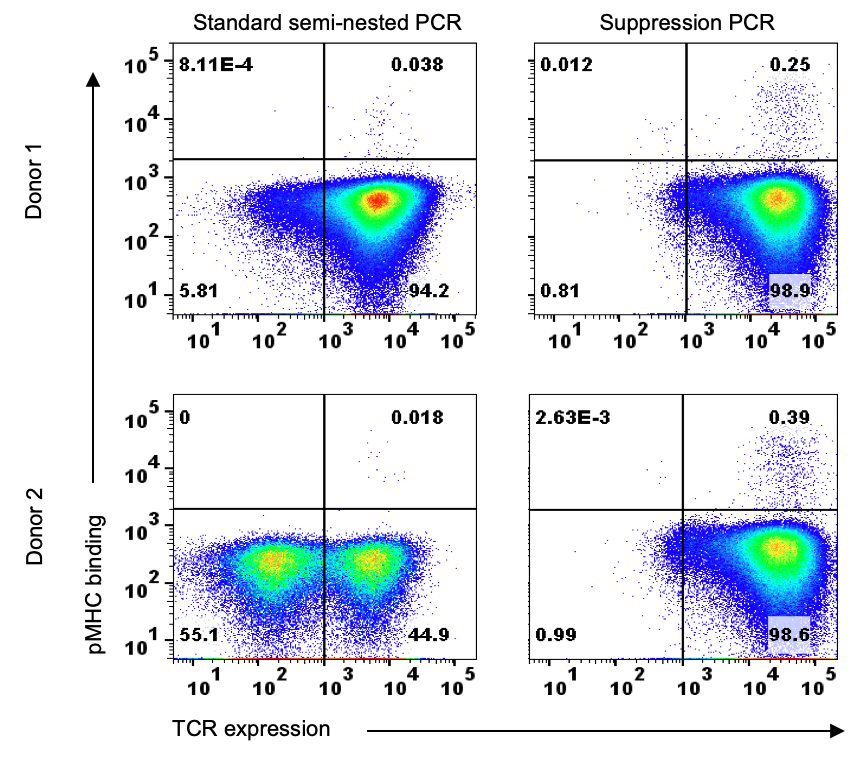


**Supplementary Figure 2. Functional analysis of TCR specificity after library generation from standard semi-nested versus suppression PCRs.** TCRα:β libraries from Donor 1 (top) and Donor 2 (bottom) were expressed in J.RT3/CD8 cells and validated for TCR expression and binding to tetrameric complexes of *RAK*/HLA-B*08:01 (top) or *TLD*/HLA-A*02:01 (bottom). TCR expression is shown on the x-axis, and pMHC binding is shown on the y-axis. Left, flow cytometry plots were newly generated in the current study. Right, flow cytometry plots were reproduced from a previous study for comparative purposes *(34)*.

**
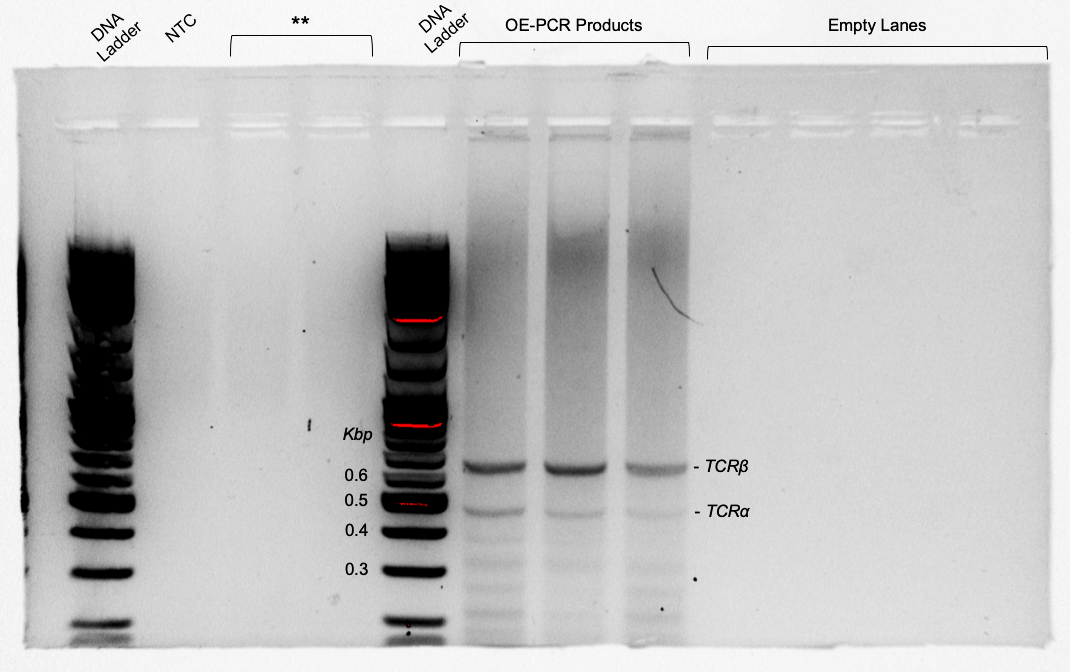
**

**
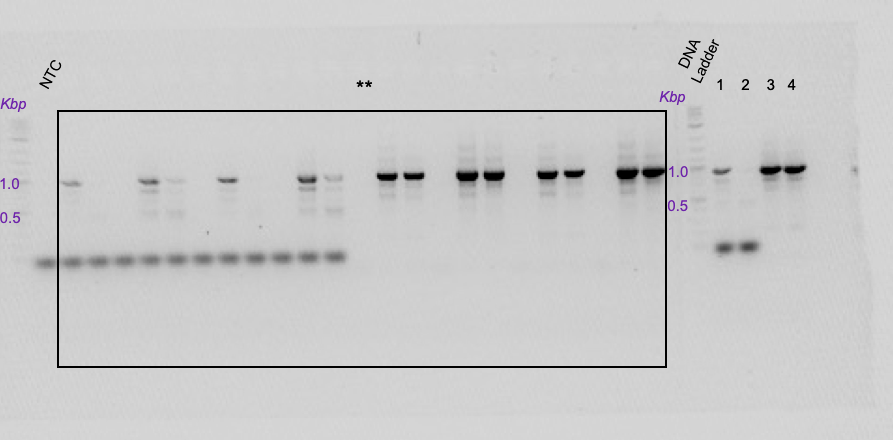
**

**Supplementary Figure 3. Agarose gel electrophoresis of amplified TCRα and TCRβ cDNAs.** Representative agarose gel electrophoresis images showing amplification of unpaired TCRα and TCRβ cDNAs via overlap extension (OE) RT-PCR (top, corresponds with Figure 4B) and a comparison of products from the first semi-nested PCR with products from various control PCRs (bottom, corresponds with Figure 4C). NTC, no-template control.


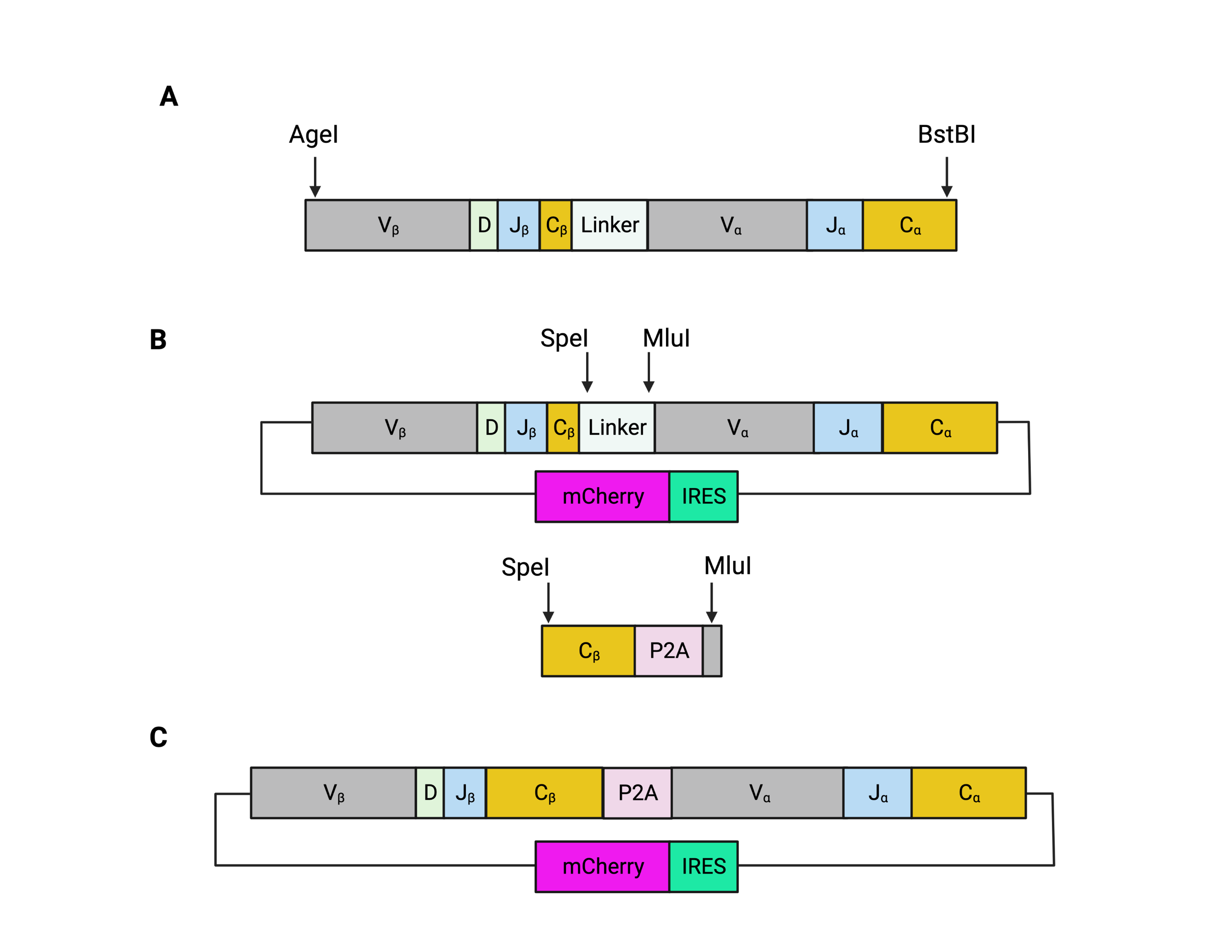


**Supplementary Figure 4. Schematic representation of the TCR library cloning pipeline.** (**A**) Natively paired TCRα:β amplicons were digested using engineered AgeI and BstBI sites in the Vβ and Cα regions, respectively. (**B**) The TCRα:β linker region was substituted with a P2A expression cassette using MluI and SpeI sites in the Cβ and Vα leader regions, respectively. (**C**) The complete TCRα:β vector ready for lentiviral transduction.


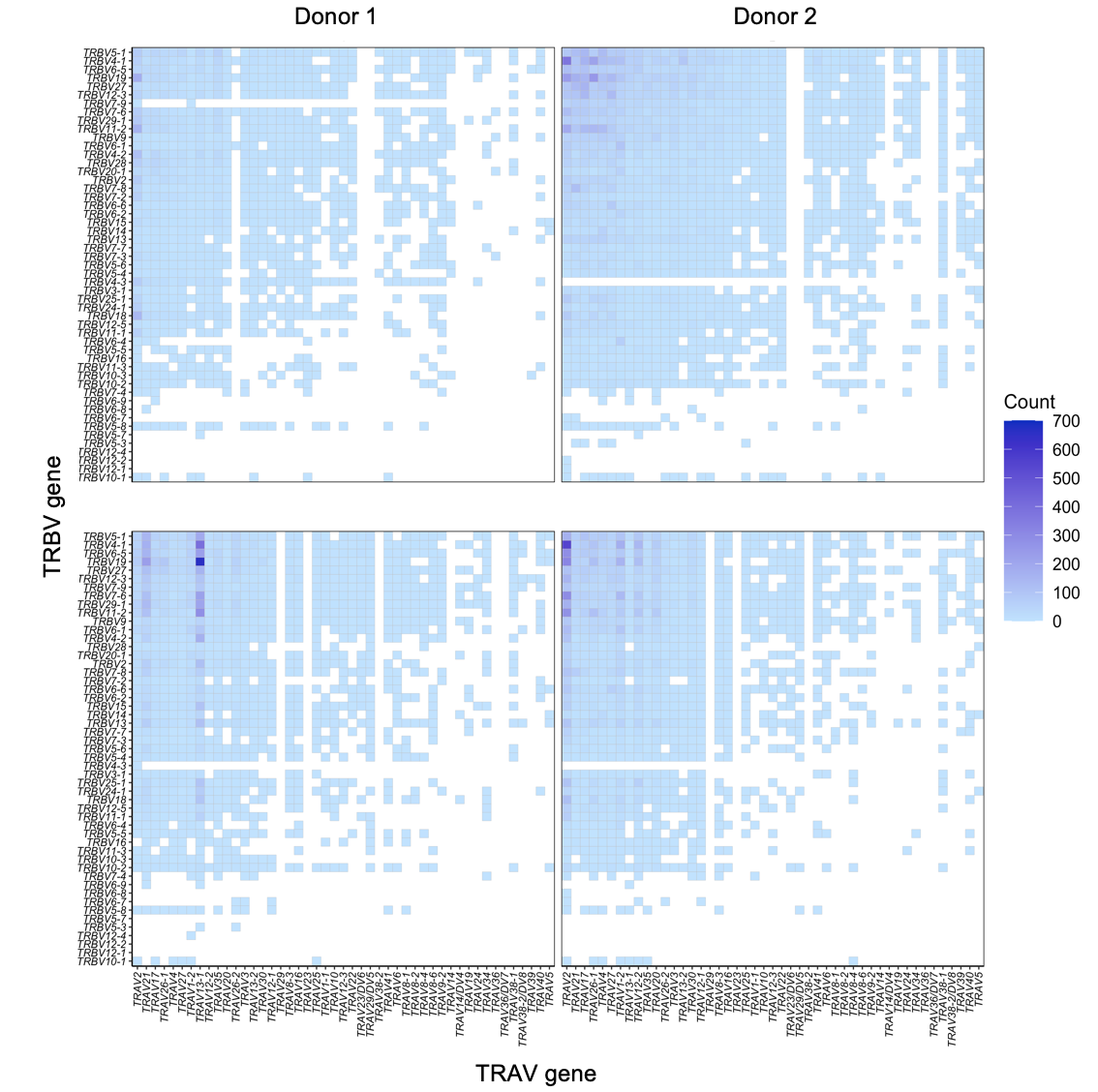


# **Supplementary Figure 5. Natively paired TCRα:β V gene use in donors with acute IM.** Heatmaps showing TCRα:β V gene use in two donors with acute IM determined using a suppression PCR (top) or a standard PCR (bottom). Top, data were reproduced from a previous study for comparative purposes *(34)*. Bottom, data were newly generated in the current study.

**A**


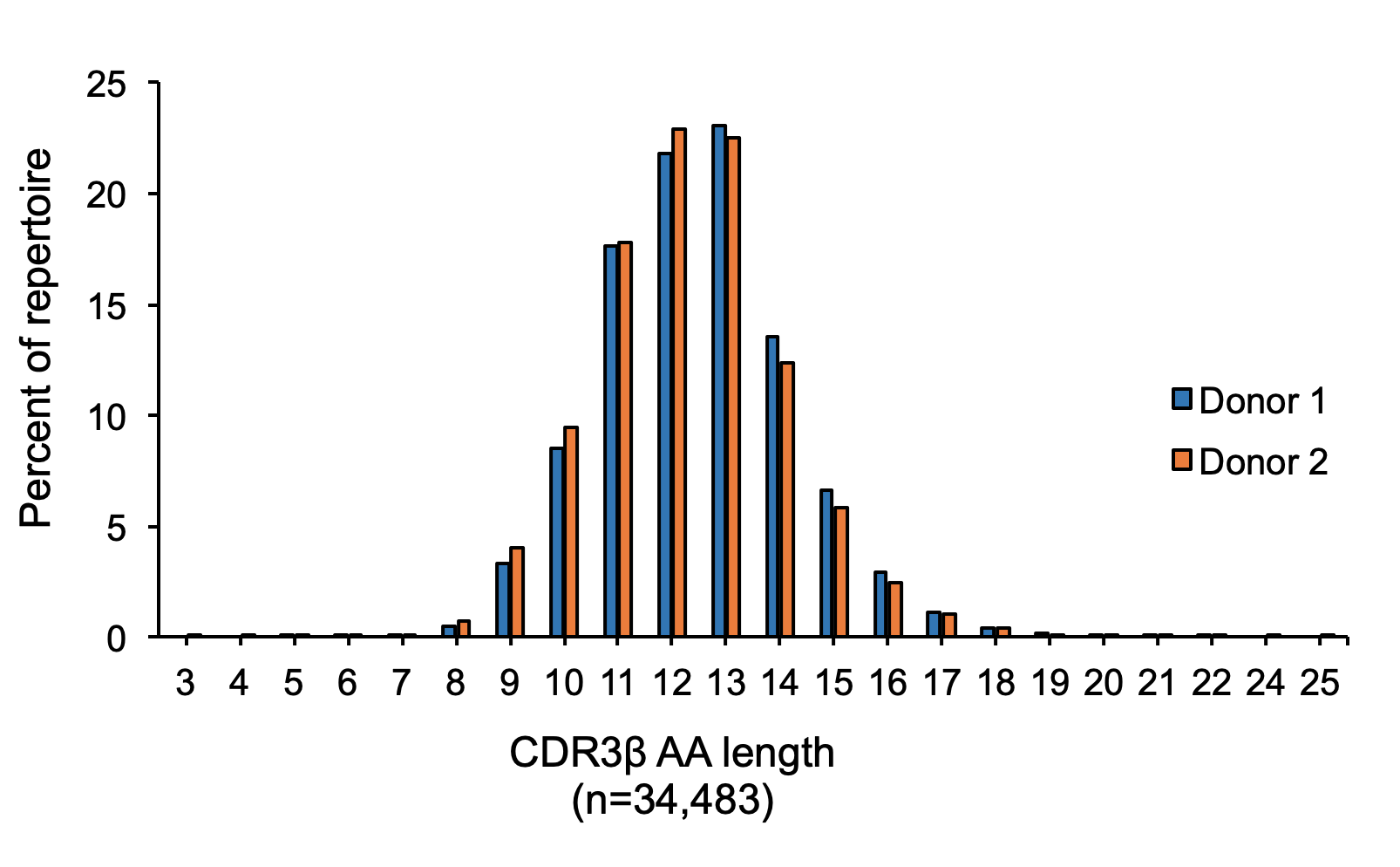


**B**


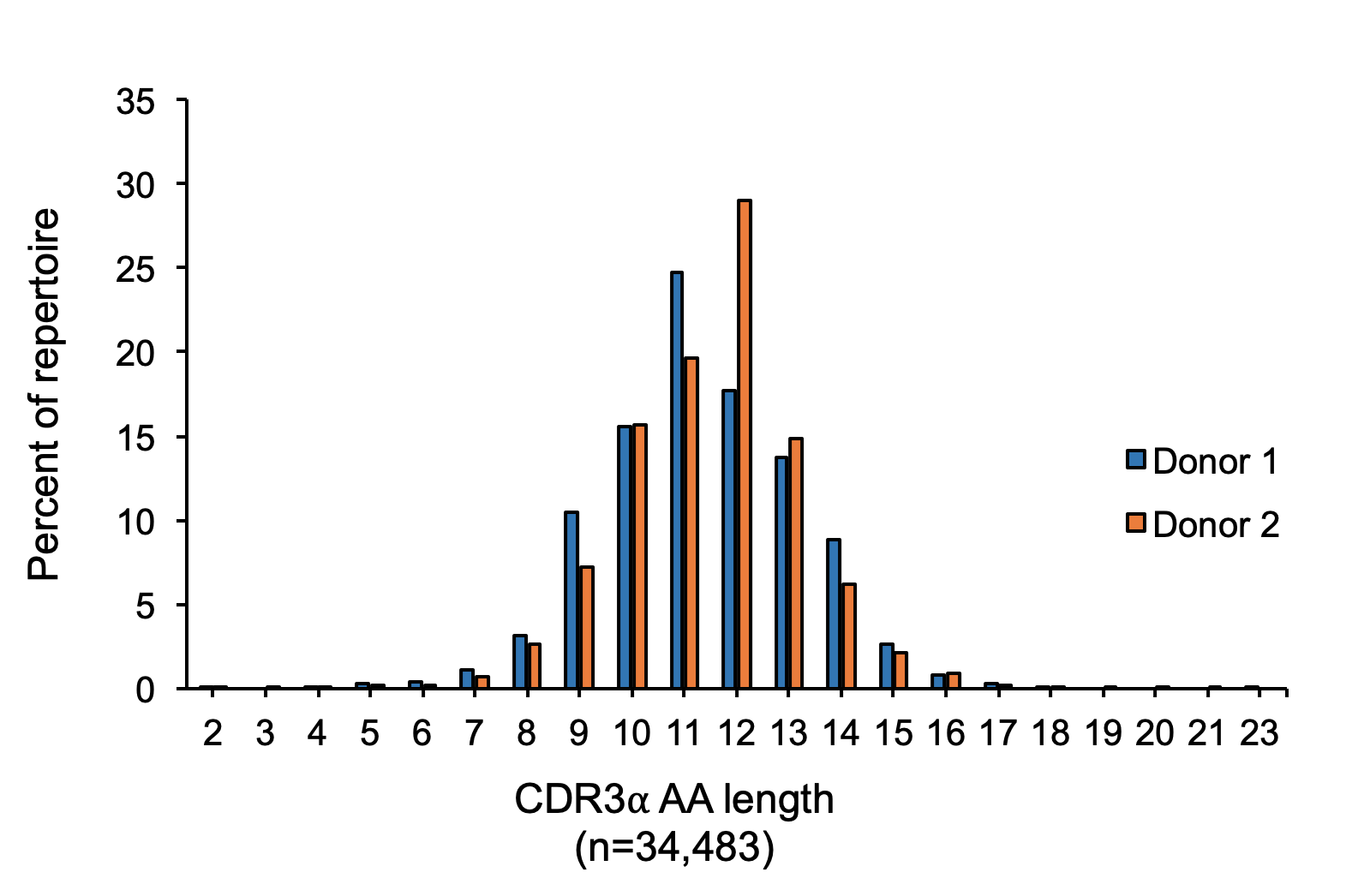


**Supplementary Figure 6. CDR3β and CDR3α length distributions in donors with acute IM.** (**A**) CDR3β amino acid length distributions in two donors with acute IM. (**B**) CDR3α amino acid length distributions in two donors with acute IM. AA, amino acid.

**SUPPLEMENTARY TABLES**

**Supplementary Table 1. Restriction enzyme site mutations introduced into the leader sequences of TRBV and TRAV genes.**

| **TRBV/TRAV gene** | **Restriction enzyme** | **Restriction site sequence** | **Nucleotide sequence pre-mutation** | **Nucleotide sequence post-mutation** |
| --- | --- | --- | --- | --- |
| TRBV15-1* | AgeI | ACCGGT | atgggtcctgggcttctccactggatggccctttgtctccttggaacaggtcatggg | atgggtcctgggcttctccactggatggccctttgtctccttggaaccggtcatggg |
| TRBV6-5 | AgeI | ACCGGT | atgagcatcggcctcctgtgctgtgcagccttgtctctcctgtgggcaggtccagtg | atgagcatcggcctcctgtgctgtgcagccttgtctctcctgtgggcaggaccggtg |
| TRBV5-6 | SpeI | ACTAGT | atgggccccgggctcctctgctgggcactgctttgtctcctgggagcaggcttagtg | atgggccccgggctcctctgctgggcactgctttgtctcctgggagcaggactagtg |
| TRBV9-1 | AgeI | ACCGGT | atgggcttcaggctcctctgctgtgtggccttttgtctcctgggagcaggcccagtg | atgggcttcaggctcctctgctgtgtggccttttgtctcctgggagcaggaccggtg |
| TRBV24-1 | AgeI | ACCGGT | atggcctccctgctcttcttctgtggggccttttatctcctgggaacagggtccatg | atggcctccctgctcttcttctgtggggccttttatctcctgggaaccggttccatg |
| TRBV30_(_*_NF_*_)_ | BsiWI | CGTACG | atgctctgctctctccttgcccttctcctgggcactttctttggggtcaga | atgctctgctctctccttgcccttctcctgggcactttctttggcgtacga |
| TRAV8-2* | MluI | ACGCGT | atgctcctgctgctcgtcccagtgctcgaggtgatttttactctgggaggaaccaga | atgctcctgctgctcgtcccagtgctcgaggtgatttttactctgggaggaacgcgt |
| TRAV27 | NheI | GCTAGC | atggtcctgaaattctccgtgtccattctttggattcagttggcatgggtgagc | atggtcctgaaattctccgtgtccattctttggattcagctagcatgggtgagc |
| TRAV40-1 | SpeI | ACTAGT | atgaactcctctctggactttctaattctgatcttaatgtttggaggaaccagc | atgaactcctctctggactttctaattctgatcttaatgtttggaggaactagt |
| TRAV18-1_(_*_NF_*_)_ | BsIWI | CGTACG | atgctgtctgcttcctgctcaggacttgtgatcttgttgatattcagaaggaccagt | atgctgtctgcttcctgctcaggacttgtgatcttgttgatattcagacgtacgagt |
| TRAV16-1_(_*_NF_*_)_ | MluI | ACGCGT | atgaagcccaccctcatctcagtgcttgtgataatatttatactcagaggaacaaga | atgaagcccaccctcatctcagtgcttgtgataatatttatactcagaggaacgcgt |
| TRAV13-2_(_*_NF_*_)_ | XhoI | CTCGAG | atggcaggcattcgagctttatttatgtacttgtggctgcagctggactgggtgagcaga | atggcaggcattcgagctttatttatgtacttgtggctgcagctggactgggtctcgaga |
| TRAV9-2_(_*_NF_*_)_ | MluI | ACGCGT | atgaactattctccaggcttagtatctctgatactcttactgcttggaagaacccgt | atgaactattctccaggcttagtatctctgatactcttactgcttggaagaacgcgt |
| TRAV17-1_(_*_NF_*_)_ | NheI | GCTAGC | atggaaactctcctgggagtgtctttggtgattctatggcttcaactggctagggtgaac | atggaaactctcctgggagtgtctttggtgattctatggcttcagctagctagggtgaac |
| TRAV38-2_(_*_NF_*_)_ | XhoI | CTCGAG | atggcatgccctggcttcctgtgggcacttgtgatctccacctgtcttgaatttagcatg | atggcatgccctggcttcctgtgggcacttgtgatctccacctgtctcgagtttagcatg |
| TRAV8-5_(_*_NF_*_)_ | BsiWI | CGTACG | atgctcctggtgctcatcccactgctggggatacattttgtcctgagaactgtcaga | atgctcctggtgctcatcccactgctg gggatacattttgtcctgcgtacggtcaga |

*, restriction enzyme site incorporated in the final primer design; *NF*, non-functional or suboptimal cloning sequence. All mutations are silent.

**Supplementary Table 2. Restriction enzyme site mutations introduced into the TRBC and TRAC genes.**

| **TRBC/TRAC gene** | **Restriction enzyme** | **Restriction site sequence** | **Original nucleotide sequence** | **Mutated nucleotide sequence** | **Mutated amino acids** |
| --- | --- | --- | --- | --- | --- |
| TRBC1 | NheI | GCTAGC | gccaca | gctagc | T28S |
| TRBC1 | SphI | GCATGC | gtgtgc | gcatgc | V30A |
| TRBC1 | NheI | GCTAGC | actggtgtg | gctagcctg | V30A |
| TRBC1 | XhoI | CTCGAG | tttgag | ctcgag | F14L |
| TRBC1*_(NF)_* | XhoI | CTCGAG | cccgag | ctcgag | P9L |
| TRBC1*_(S)_** | SpeI | ACTAGT | acactggtg | acactagtg | - |
| TRAC*_(S)_** | BstBI | TTCGAA | gactctaaa | gattcgaaa | - |

Colors indicate target bases (blue) and mutated bases (red). *, restriction enzyme site incorporated in the final primer design; *NF*, non-functional or suboptimal cloning sequence; *S*, silent mutation.

**Supplementary Table 3. Restriction enzyme site modification attempts that produced suboptimal pMHC binding and TCR expression after cloning and validation via FACS.**

| **Leader sequence/ restriction site** | **% pMHC binding** | **% TCR expression** |
| --- | --- | --- |
| TRAV16-1 / MluI | 5.53 | 13.1 |
| TRBCP9L / XhoI | 46.3 | 49.2 |
| TRBCA19L / XhoI | 1.62 | 0.63 |

**Supplementary Table 4. NGS-based quantitative analysis of antigen-specific TCRs.**

| **CDR3β sequence** | **Paired TCRα:β library** | |  | ***RAK* Rd1 sort** | | |
| --- | --- | --- | --- | --- | --- | --- |
|  | Fraction | Reads |  | Fraction | Reads | ER |
| CASSPLTGELFF | 0.00359 | 2,046 |  | 0.778 | 417,043 | 217 |
| CASSWGTGENTEAFF | 0.000116 | 66 |  | 0.00141 | 754 | 12.2 |

Antigen specificity was confirmed via binding to tetrameric complexes of *RAK*/HLA-B*08:01. The selected CDR3β sequences were identified in a previous study of natively paired TCRα:β gene libraries from the same donor *(34)*.
